# Critical dependence of magnetostructural coupling and magnetocaloric effect on particle size in Mn-Fe-Ni-Ge compounds

**DOI:** 10.1038/srep20993

**Published:** 2016-02-17

**Authors:** Rongrong Wu, Feiran Shen, Fengxia Hu, Jing Wang, Lifu Bao, Lei Zhang, Yao Liu, Yingying Zhao, Feixiang Liang, Wenliang Zuo, Jirong Sun, Baogen Shen

**Affiliations:** 1 Beijing National Laboratory for Condensed Matter Physics and State Key Laboratory of Magnetism, Institute of Physics, Chinese Academy of Sciences, Beijing 100190, P. R. China; 2 High Magnetic Field Laboratory, Chinese Academy of Sciences, Hefei 230031, P. R. China

## Abstract

Magnetostructural coupling, which is the coincidence of crystallographic and magnetic transition, has obtained intense attention for its abundant magnetoresponse effects and promising technological applications, such as solid-state refrigeration, magnetic actuators and sensors. The hexagonal Ni_2_In-type compounds have attracted much attraction due to the strong magnetostructural coupling and the resulted giant negative thermal expansion and magnetocaloric effect. However, the as-prepared samples are quite brittle and naturally collapse into powders. Here, we report the effect of particle size on the magnetostructural coupling and magnetocaloric effect in the Ni_2_In-type Mn-Fe-Ni-Ge compound, which undergoes a large lattice change across the transformation from paramagnetic austenite to ferromagnetic martensite. The disappearance of martensitic transformation in a large amount of austenitic phase with reducing particle size, to our best knowledge, has not been reported up to now. The ratio can be as high as 40.6% when the MnNi_0.8_Fe_0.2_Ge bulk was broken into particles in the size range of 5~15 μm. Meanwhile, the remained magnetostructural transition gets wider and the magnetic hysteresis becomes smaller. As a result, the entropy change drops, but the effective cooling power *RC*_*effe*_ increases and attains to the maximum at particles in the range of 20~40 μm. These observations provide constructive information and highly benefit practical applications for this class of novel magnetoresponse materials.

An increasing attention has been attracted to magnetic refrigeration technique based on magnetocaloric effect (MCE) because of environmental concerns and energy savings. Since the discovery of MCE by P. Weiss and A. Piccard in 1917^1^,^2^, lots of efforts have been dedicated to theoretical and experimental investigations. In particular, renewed interest arouses due to the discovery of giant MCE relative to magnetostructural transitions. The well-known materials include Gd_5_(Si,Ge)_4_^3^, La(Fe,Si)_13_^4^,^5^, MnFeP_1−x_As_x_^6^, and NiMn-based Heusler alloys^7^,^8^,^9^,^10^, where the magnetic phase transition always takes place along with a discontinuous change in lattice parameters and/or crystal symmetry. Generally, the magnetostructural coupling can be explained by a strong dependence of the exchange constant on interatomic distance, which introduces mutual dependencies between the lattice and spin ordering. As a result, materials with such novel characteristics often exhibit abundant magnetoresponses, such as magnetic-field-induced strains^11^ and MCE^7^,^8^,^9^,^10^, as well as negative thermal expansion (NTE) behavior^12^.

MM’X (M, M’ = transition metals, X = Si, Ge, Sn) compound with hexagonal Ni_2_In-type structure is another material that attracted much attention due to the strong magnetostructural coupling^13^,^14^,^15^,^16^. The optimized compositions with concurrent magnetic and structural transitions have been discovered showing giant negative thermal expansion^14^ and magnetocaloric effect^15^. However, the stoichiometric MnNiGe, a member of MM’X family, does not show magnetostructural coupling. It undergoes a martensitic structural transformation from Ni_2_In-type austenite to TiNiSi-type martensite at *T*_*stru*_~470 K, and a separate magnetic transition at lower temperature of *T*_*N*_^*M*^~346K. Due to the different structural symmetry across the *T*_*stru*_, the martensitic and austenitic phases display different magnetic structure. The martensite has spiral antiferromagnetic (AFM) structure with Neel temperature *T*_*N*_^*M*^ at 346K while the austenite shows ferromagnetic (FM) structure with intrinsic Curie temperature *T*_*C*_^*A*^ at a lower temperature of 205K^17^. The AFM coupling in the martensitic phase is not robust. The investigations carried out by Liu *et al*.^16^ revealed that the substitution of Mn, Ni by Fe atoms can convert the AFM into FM, and enhance the stability of austenitic phase. As a result, the *T*_*stru*_ shifts to low temperature, and magnetostructural transition, *T*_*mstru*_, and large MCE have been realized in a very wide temperature window from 350 to 70K.

Generally, strong magnetic volume effect is a common feature for a material with magnetostructural coupling. The materials are usually brittle and even naturally collapse into powders during preparation. People eagerly want to know what about the performance for the small particles. Moore *et al*. studied Gd_5_Ge_4_ material and found that the operating field for phase transition becomes lower with reducing particle size due to the reduction of internal strain^18^. Lyubina *et al*. studied the performance of La(Fe,Si)_13_ system with porous architecture, and found that magnetic hysteresis can be improved due to partial removal of grain boundaries that restrains volume expansion, and excellent performance can be maintained^19^. Moreover, Kruk *et al*. studied grain-size-dependent magnetic properties in nanocrystalline Gd^20^, the well-known elemental metal showing large MCE due to second-order magnetic transition, and found that the magnetic and electronic structure of the atoms in the grain boundaries differs distinctively from that in the grain interiors. This work demonstrated the notable effect of the introduced defects on the structure and magnetic properties.

Here, we report the particle size effect of magnetostructural coupling and MCE in the hexagonal Ni_2_In-type Mn-Fe-Ni-Ge compounds. This class of materials is particularly unique, whose magnetostructural transition is sensitive to pressure rather than magnetic field^15^,^21^. In the process of pulverization, residual strain and defects are unavoidably introduced and interior stress in the grain and grain boundaries will be re-distributed, which largely affect the magnetostructural coupling. Our studies reveal distinct difference compared to other giant MCE materials. With reducing particle size, a large amount of austenitic phase loses the martensitic transformation, i.e. magnetostructural transition, and retains the hexagonal FM structure in the entire temperature range. Although the entropy change drops, the effective cooling power (RCP) increases by 22% as the bulk was broken into particles in the size range of 20~40 μm.

## Results

Magnetization measurements indicated that the prepared Mn_1−x_Fe_x_NiGe and MnNi_1−y_Fe_y_Ge bulk show magnetostructural transition at Fe concentration 0.08 ≤ x ≤ 0.26, 0.20 ≤ y ≤ 0.30, consistent with previous report^16^. Typically, we chose Mn_0.82_Fe_0.18_NiGe and MnNi_0.8_Fe_0.2_Ge to study the particle size effect, whose *T*_*mstru*_ locates at 198K and 302K, respectively.

**Figure 1:**
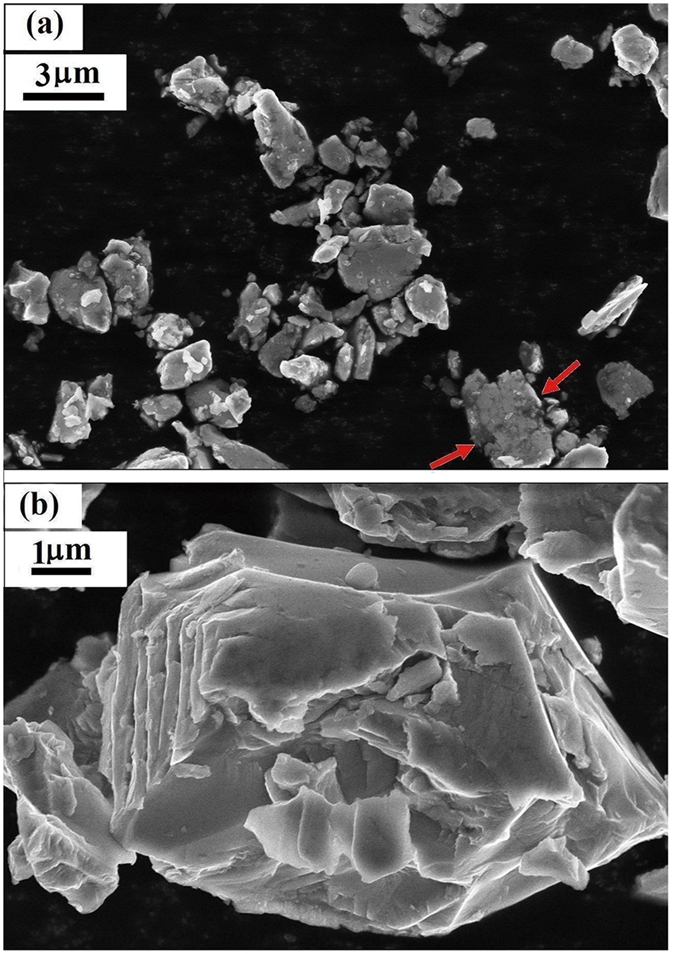
The SEM micrograph of particles (**a**) P6(1~3 μm), and (**b**) one particle of P4(5~10 μm) for Mn_0.82_Fe_0.18_NiGe.

Samples derived from the same ingot were manually ground into irregular powders using an agate mortar by hand protected by acetone and Ar atmosphere, and the particle size was controlled by the milling time. For MnNi_0.8_Fe_0.2_Ge, two samples with different sizes were made and namely S1(20~40mm) and S2(5~15mm), while for Mn_0.82_Fe_0.18_NiGe, six samples namely P1(60~100mm), P2(20~40mm), P3(10~20mm), P4(5~10mm), P5(2~5mm), and P6(1~3mm). A piece of single fragment from the same ingot was chosen as bulk for comparison. The micrographs of these powders were examined under scanning electron microscope (SEM), and the details of typical samples can be found in Fig. 1. One can notice that some particles of P6(1~3 μm) involves repeated fracturing and cold welding with particles smaller than 1 μm (see the parts indicated by red arrows in Fig. 1a), while the particles of P4(5~10 μm) are composed of layers. These results evidence the possible introduction of stress and defects during the pulverization process.

**Figure 2:**
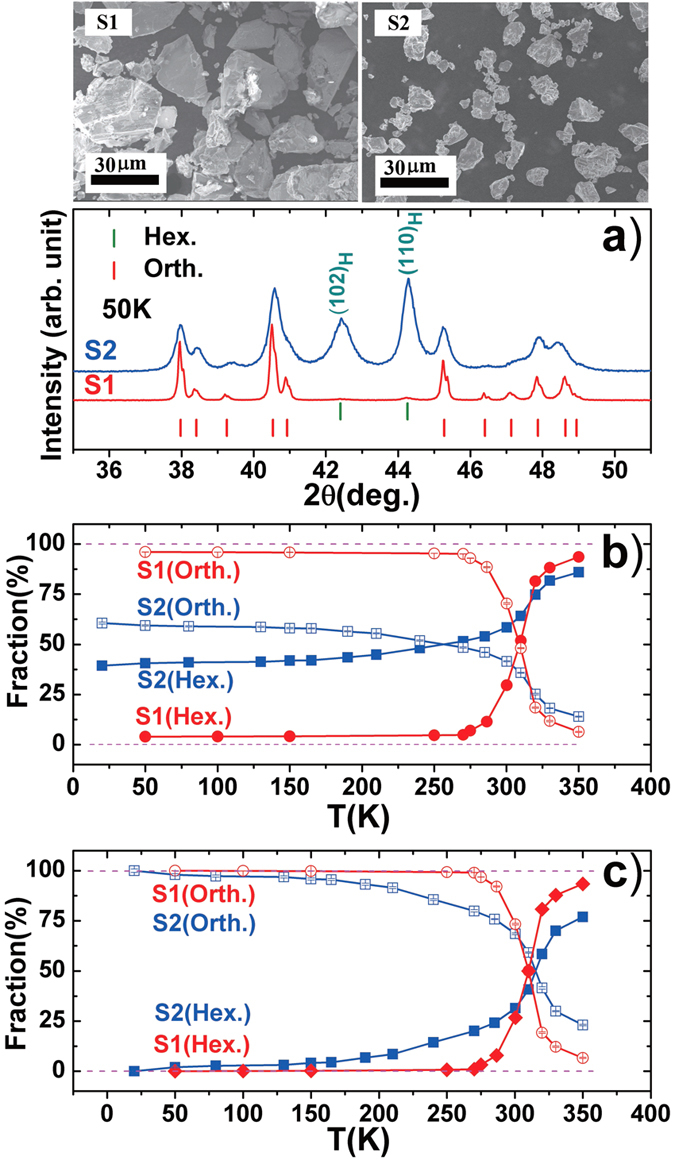
The compared (**a**) XRD patterns collected at 50 K, (**b**) fractions of hexagonal and orthorhombic phases as a function of temperature, and (**c**) deduced results from (**b**) after deducting the fraction that lost magnetostructural transformation, for particles S1 (20~40 μm) and S2 (5~15 μm) of MnNi_0.8_Fe_0.2_Ge. For the intuition purpose, the morphology of the corresponding samples was given on the top of (**a**).

To examine the change of magnetostructural transition with particle size, we performed variable temperature x-ray diffraction (XRD) measurements for different samples. Typically, Fig. 2a presents the XRD patterns collected at 50K for S1(20~40 μm) and S2(5~15 μm) of MnNi_0.8_Fe_0.2_Ge. Distinct difference can be identified. The particles S1(20~40 μm) shows almost single phase of orthorhombic structure while the S2(5~15 μm) clearly displays the coexistence of orthorhombic and hexagonal structure, noting the appearance of high (102)_H_ and (110)_H_ peaks of hexagonal phase. For the intuition purpose, the morphology of the corresponding samples was given on the top of Fig. 2a. Based on Rietveld refinements with 2θ from 20 to 70 degree, the calculated ratio of hexagonal phase is about 40.6% and 4.0% at 50K for S2(5~15 μm) and S1(20~40 μm), respectively. Figure 2b displays the fractions of hexagonal and orthorhombic phases as a function of temperature covering the phase transition for the both samples. The sample with small particles S2(5~15 μm) remains high ratio of hexagonal phase in the entire temperature range, and the ratio increases from 40.6% to 51.6% as the temperature increases from 50K to 270K, indicating that at least 40.6% of sample loses the magnetostructural transition. Meanwhile, the remained magnetostructural transition becomes broadening as the bulk was broken into S2(5~15 μm). For the large particles S1(20~40 μm), nearly unchanged ratio (<5%) of hexagonal phase appears from 50K to 270K, indicating that less than 5% sample loses the magnetostructural transformation, and the remained magnetostructural transformation is still sharp. For clarity, Fig. 2c shows the deduced results from Fig. 2b after deducting the fraction that lost magnetostructural transformation. One can notice that the phase transition width of S2(5~15 μm) is much larger than that of S1(20~40 μm) noting the coexistent region of orthorhombic and hexagonal structure can be as wide as 300K from 50K to 350K for the former, while the corresponding region is about 80K from 270K to 350K for the latter in the measured temperature range. Such behavior can be ascribed to the distribution of interior stress caused by milling, which gives rise to a distribution of the coexistence range of orthorhombic and hexagonal phases, thus broadening the transition width.

**Figure 3:**
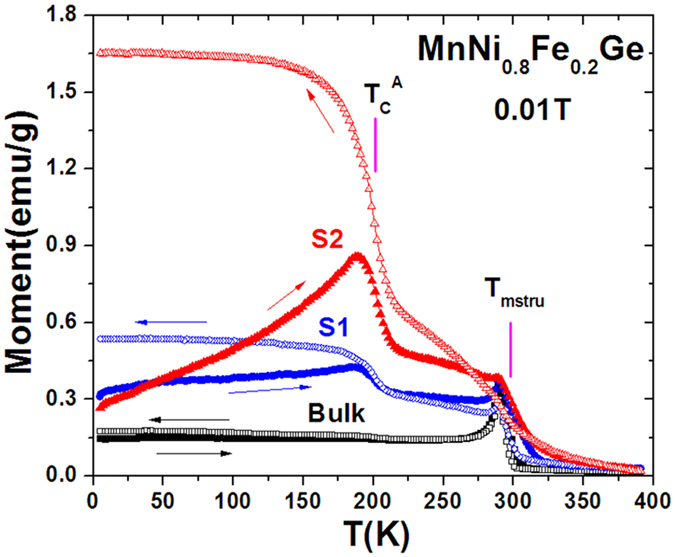
The temperature dependent magnetization (M-T curve) of particles S1(20~40 μm) and S2(5~15 μm) of MnNi_0.8_Fe_0.2_Ge measured using ZFC/FC mode under 0.01T compared to the bulk. The arrows indicate the cooling/warming paths.

Figure 3 presents the temperature dependent magnetization (M-T curve) of S1(20~40 μm) and S2(5~15 μm) measured using ZFC/FC mode under a magnetic field of 0.01T compared to the bulk. The relative low magnetization of the bulk in the low-temperature region indicates that the tilting AFM state is dominant^16^, which is similar to the stoichiometric MnNiGe^17^. The followed sharp jump is typical for a magnetic transition from AFM to FM state on heating. Upon further warming, the bulk undergoes a transition from FM to paramagnetic (PM) state at ∼302K with a thermal hysteresis of ~5K, evidencing the occurrence of first-order magnetostructural transition at *T*_*mstru*_~302K. As the bulk is broken into S1(20~40 μm) and S2(5~15 μm), we surprisingly find that one more magnetic phase transition develops at the nearly same *T*_*C*_^*A*^~200K and it becomes pronounced with reducing the particle size. Meanwhile, the position of *T*_*mstru*_ keeps nearly unchanged, and the phase transition width around the *T*_*mstru*_ becomes broadening. These results agree well with the XRD observations in Fig. 2. Undoubtedly, the emerging new transition at *T*_*C*_^*A*^~200K should be the FM ordering temperature of the fractions of austenite that lost the martensitic structural transition. In other words, this fraction of austenite phase retains the hexagonal structure in the entire temperature range without any structural transition. The separation between ZFC and FC magnetization below the *T*_*C*_^*A*^ is related to the pinning of FM spin structure by AFM domains due to the coexistence of AFM and FM clusters^22^.

**Figure 4:**
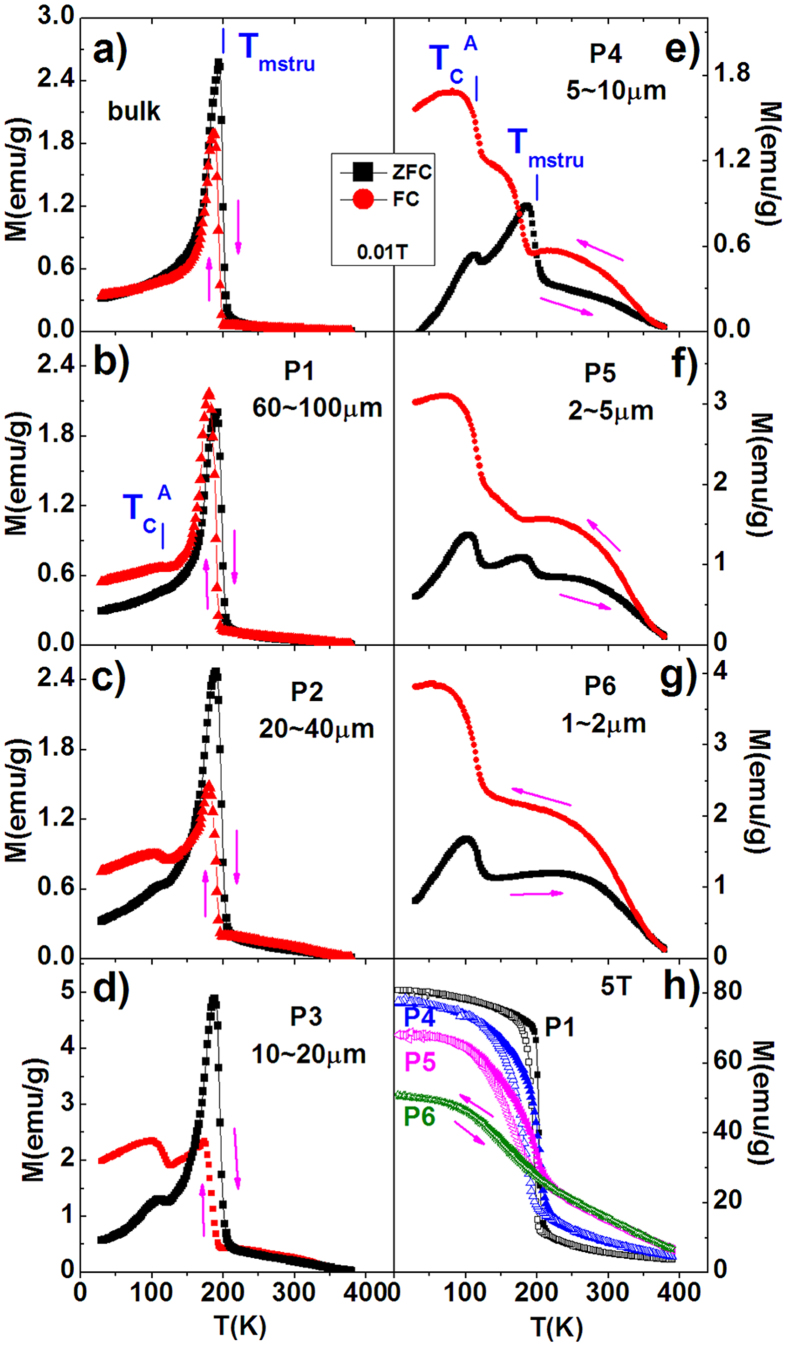
ZFC/FC magnetization as a function of temperature (M-T curve) under 0.01T for (**a**) the bulk, (**b**) P1(60~100 μm), (**c**) P2(20~40 μm), (**d**) P3(10~20 μm), (**e**) P4(5~10 μm), (**f**) P5(2~5 μm), and (**g**) P6(1~3 μm) of Mn_0.82_Fe_0.18_NiGe. (**h**) presents the M-T curves under 5T for typical samples. The arrows indicate the cooling/warming paths.

Such a phenomenon is extremely unique in the materials with magnetostructural transition. To confirm this is a universal behavior, we further chose Mn_0.82_Fe_0.18_NiGe with substitution of Mn by Fe and investigated the particle size effect. Figure 4 shows the ZFC/FC magnetization as a function of temperature measured under 0.01T for P1(60~100mm), P2(20~40mm), P3(10~20mm), P4(5~10mm), P5(2~5mm), and P6(1~3mm) in comparison to the bulk. Similar to MnNi_0.8_Fe_0.2_Ge, the replacement of Mn by Fe can also be able to shift the *T*_*stru*_ to low temperature, and the concurrent FM-PM and structural transition take place around *T*_*mstru*_~198K for the bulk of Mn_0.82_Fe_0.18_NiGe. Amazingly also, one more magnetic phase transition develops at a low temperature around *T*_*C*_^*A*^~116K, and becomes pronounced and dominant with reducing particle size. Meanwhile, the magnetostructural transition around the *T*_*mstru*_~198K becomes trivial and even disappears as the particle approaches to P6(1~3 μm) (Fig. 4g). These results indicate that a large fraction of austenite phase that lost the martensitic structural transition also appears, and the remained magnetostructural transition window gets broadening. The growing magnetic transition at *T*_*C*_^*A*^~116K should be the FM ordering of austenite phase in Mn_0.82_Fe_0.18_NiGe. It appears at a much lower temperature compared to the *T*_*C*_^*A*^ (~205K) of stoichiometric MnNiGe^17^. This result indicates that the replacements of Mn by Fe largely affects the FM coupling in the hexagonal structure, noting the Mn atoms are the main carriers of magnetic moments in the MnNiGe compounds^17^. In contrast, the *T*_*C*_^*A*^ (~200K) in MnNi_0.8_Fe_0.2_Ge is very close to the one of stoichiometric MnNiGe (*T*_*C*_^*A*^~205K), indicating that the FM interaction in the hexagonal structure keeps nearly unchanged upon the substitution of Ni by Fe atoms in MnNi_1−y_Fe_y_Ge, noting the Ni atoms contribute a little to the molecular moment^17^. Figure 4h presents the M-T curves measured under a high magnetic field of 5T for the samples with different particles of Mn_0.82_Fe_0.18_NiGe. Full FM behavior appears noting the AFM coupling at low temperature has been converted into FM interaction under 5T. The phase transition becomes broadening and the magnetization at 5K (approximately the saturated magnetization, *M*_*S*_) notably decreases with reducing the particle size. Compared to P1(60~100 μm), the *M*_*S*_ of P6(1~3 μm) reduces by 37%, which can be understood considering two aspects. One is the possible change of occupations of magnetic atoms caused by the introduced defects during the process of pulverization^20^. Another is the notable increase of the fraction of hexagonal phase that lost the martensitic transformation, noting the hexagonal phase may have a smaller magnetic moment than the orthorhombic phase in the Fe-doped MnNiGe with the conversion from AFM to FM^16^, similar to the case in MnCoGe-based alloys^23^.

**Figure 5:**
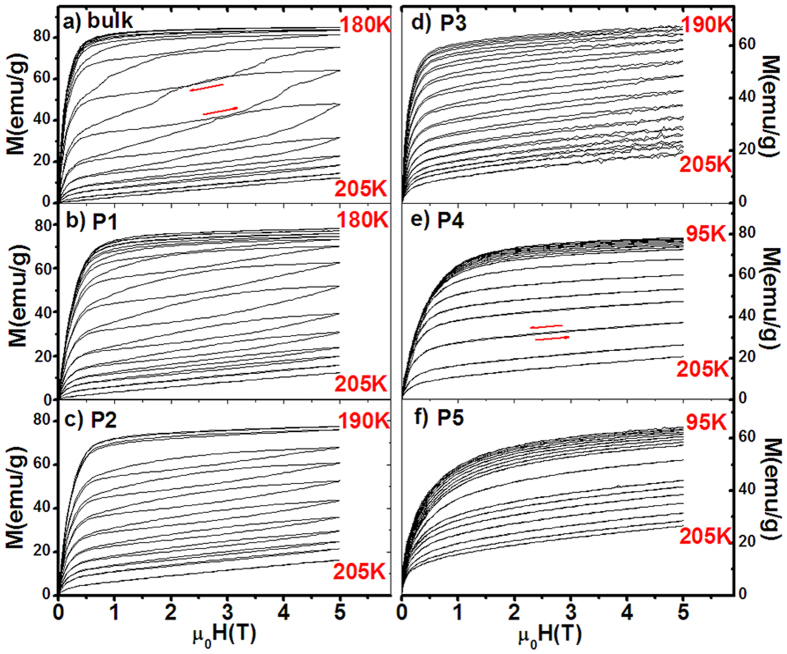
The isothermal magnetization (M-H curves) of the particles P1(60~100mm), P2(20~40mm), P3(10~20mm), P4(5~10mm), and P5(2~5mm) compared to the bulk for Mn_0.82_Fe_0.18_NiGe. The arrows indicate ascending/descending paths of magnetic field.

**Figure 6:**
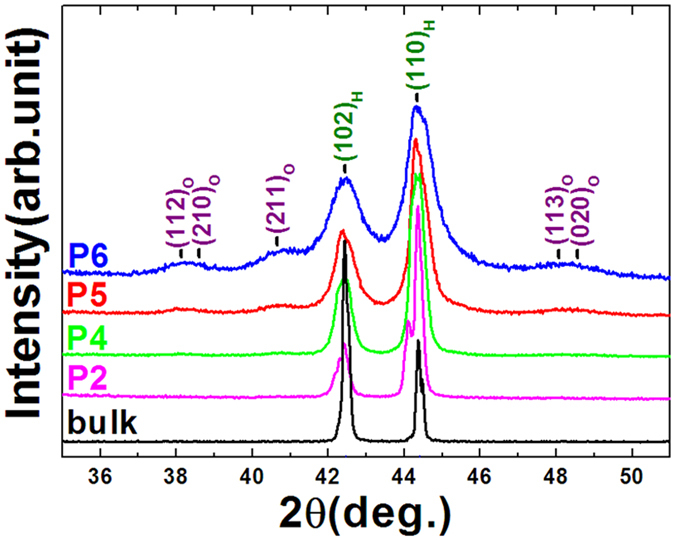
XRD patterns collected at room temperature for the typical particles P2(20~40mm), P4(5~10mm), P5(2~5mm), and P6(1~3mm) compared to the bulk for Mn_0.82_Fe_0.18_NiGe.

Furthermore, to examine the performance of MCE with various particle sizes, we measured the isothermal magnetization (M-H curves) of the particles compared to the bulk for Mn_0.82_Fe_0.18_NiGe (Fig. 5). Considerable magnetic hysteresis appears in the bulk, which rapidly gets suppressed with reducing particle size, and even disappears as the particles approach to P4(5~10 μm). The maximal hysteresis loss up to 5T is 42.7, 27.2, 18.5, 9.0, 0, 0, and 0 J/kg for the bulk, P1(60~100 μm), P2(20~40 μm), P3(10~20 μm), P4(5~10 μm), P5(2~5 μm), and P6(1~3 μm), respectively. The notable reduction of hysteresis loss can be ascribed to two factors. One is the growing of the hexagonal phase that lost structural transition. This fraction of sample shows second-order transition around the *T*_*C*_^*A*^ and does not have any hysteresis in nature. Another is the notably increased surface area of sample and the fundamental changes of strain distribution and grain boundaries with reducing particle size, which notably improves heat transfer and reduces the hysteresis loss^24^. Moreover, the M-H curves at paramagnetic austenitic region (such as 205K) become gradually bent with reducing the particle size, indicating more and more FM martensitic phase appears in the paramagnetic region. This result accords well with the XRD performance collected at room temperature for the samples with different particles (Fig. 6), where the typical peaks (112)_O_, (211)_O_, (113)_O_ of orthogonal structure grow up in the background of hexagonal structure. This interesting feature further evidences the broadening of magnetostructural transition with reducing particle size.

**Figure 7:**
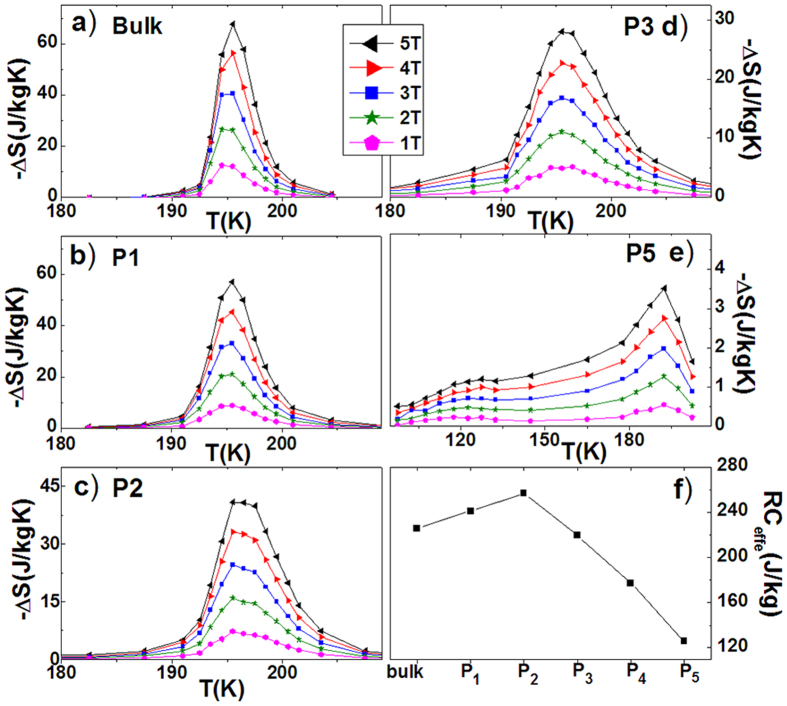
Entropy change as functions of temperature and magnetic fields for (**a**) the bulk, (**b**) P1(60~100 μm), (**c**) P2(20~40 μm), (**d**) P3(10~20 μm), and (**e**) P5(2~5 μm) of Mn_0.82_Fe_0.18_NiGe. (**f**) presents the effective *RC*_*effe*_ for the different samples.

Based on the isothermal M-H curves, we calculated the magnetic entropy change, Δ*S*, using Maxwell relation. The maximal *|ΔS|* is 67.5, 57.0, 40.8, 28.0, 10.5, 3.5J/kgK, and the effective cooling power *RC*_*effe*_ (after deducting the maximal hysteresis^25^) is 227.3, 246.4, 267.1, 215.0, 177.2, 129.0 J/kg, for the bulk, P1(60~100 μm), P2(20~40 μm), P3(10~20 μm), P4(5~10 μm), P5(2~5 μm), respectively. The results are shown in Fig. 7 for typical samples. Although the *|ΔS|* value shows a monotonous decrease with reducing particle size, the effective *RC*_*effe*_ increases and attains to the maximum at P2(20~40 μm) (Fig. 7f) due to the notable reduction of hysteresis loss (Fig. 5). The increased ratio of *RC*_*effe*_ reaches 22% noting the maximal hysteresis loss reduces by 57% when the bulk is broken into P2(20~40 μm). With further reducing the particle size, both the *|ΔS|* and *RC*_*effe*_ largely decrease because a large amount of samples lost the martensitic transition. This fraction of sample retains the hexagonal structure in the entire temperature range and shows second-order FM ordering transition around *T*_*C*_^*A*^∼116K(Fig. 4). Typically, the *ΔS* was measured around the both *T*_*C*_^*A*^ and *T*_*mstru*_ for P5(2~5 μm)(Fig. 7e). Two *ΔS* peaks appear as expected, but the maximal *|ΔS|* around the *T*_*C*_^*A*^ is only 1.2 J/kgK(5T) due to the second-order nature of magnetic transition.

## Discussion

For the origin of particle size effect, we can understand it by considering the change of interior stress and the possibly introduced residual strain and defects during the pulverization process. Actually, the effect of interior stress and/or particle size on the microstructures in solid materials has been early studied both theoretically and experimentally. In materials with a fine-scale microstructure, the stresses from grain boundaries give rise to internal stress fields that depend on the geometry. It has been well established that the lattice parameters of small metallic particles decrease with the reduction in size, which can be explained using the theoretical treatment^26^ in terms of a surface stress. Moreover, at the interfaces between two phases, the most general deformation involves independent displacements of the phases. The interface stresses can be closely related to the different strains in the two phases. Additionally, solids can also contain stresses arising from other sources, such as the constraints from the second interfacial stress or from coherency constraints and from volume misfit between the individual microstructure^27^. Hence, the pressure of the individual phase is quite different with each other noting it is not related in a simple way to the interface stress and to the curvature related to the geometry or other factors.

For the present hexagonal MM’X materials, the difference in phase volume across phase transition is particularly large, 2.7~4.0%^14^,^16^. Accordingly, the different grains of the two phases suffer quite different stresses. As the bulk was broken into small particles, the stress environments that maintain the martensitic structural transformation has been changed and most of the constraints from the second interfacial stress might be released. As a result, earthshaking changes take place in the surroundings of the grain and grain boundaries. A unique feature of the present materials is their high sensitivity to stress. It has been found that an applied hydrostatic pressure can largely shift the magnetostructural transition to lower temperature^15^,^21^ at a rate of 10K/kbar for Mn_0.93_Cr_0.07_CoGe, and a purposely introduced residual strain in a thin slice of MnCoGe_1−x_In_x_ can also be able to induce the possible appearance of a considerable amount of hexagonal phase that lost the martensitic structural transformation while the grain size keeps nearly unchanged^28^. All these demonstrate the key role of the change of interior stress and/or the introduced residual strain on the evolution of magnetostructural transition. For the present samples, although the visual size under SEM exceeds 1 μm even for P6(1~3 μm), the particle agglomeration and cold welding (Fig. 1) are actually unavoidable noting the milling time is as long as 6h for P6(1~3 μm). Thus it is possible that the actual size may be much smaller, lying in the submicron and even nanometer range. We roughly estimated the grain size from the XRD patterns shown in Fig. 6 for the typical particles P4(5~10mm) and P6(1~3 μm), and found the grain size is about 80nm and 10 nm, respectively. In this case the introduced stress and the change of interior stress during milling process can be spread into lattice inside the particles, affecting the most of materials. The response of crystallographic change to pressure is to stabilize the phase with smaller volume, which is the austenite phase in our case, eventually leading to the shift or even disappearance of the martensitic transformation.

In summary, we studied the evolution of magnetostructural coupling with particle size in the novel Mn-Fe-Ni-Ge compounds that show abundant magnetoresponse effects. Our results explicitly demonstrate that a large amount of austenite will lose the martensitic transformation with reducing the particle size. This amazing phenomenon can be closely related to the fundamental changes of stress distribution and the possibly introduced residual strain during milling process, as well as the high sensitivity of the magnetostructural transition to stress. Although the entropy change decreases with reducing the particle size, the effective cooling power *RC*_*effe*_ increases by 22% as the bulk was broken into particles in the size range of 20~40 μm due to the notable reduction of hysteresis loss. Such a tunable magnetostructural coupling with particle size is particularly unique for this class of materials. We anticipate that the present study can inspire further interest in exploring stress or particle size regulated properties, such as the NTE behavior and various magnetoresponse effects, in the novel materials and promote their abundant applications.

## Methods

### Sample preparation, magnetic measurements, and structural analysis

Mn-Fe-Ni-Ge alloys were prepared by repeatedly arc-melting appropriate amounts of starting materials in high-purity argon atmosphere (99.996%) with a base pressure of 10^−4^Pa. The commercial purities of Mn, Fe, Ni, Ge are 99.9 wt%, 99.9 wt%, 99.99 wt%, and 99.999 wt%, respectively. The obtained ingots were wrapped separately with Mo foil and subsequently homogenized in a sealed quartz tube under vacuum of 10^−4^ Pa at 875   oC for 6 days, then quenched in liquid nitrogen. Magnetic measurements were performed using a superconducting quantum interference device magnetometer (SQUID-VSM). The temperature rate is 5K/min for the measurements of M-T curves shown in Figs 3 and 4, while the sweep rate of magnetic field is 50 Oe/s for the M-H curves shown in Fig. 5. The samples are brittle. We usually chose an irregular single fragment with length/width ratio about 2 for magnetic measurements. If we take the cylinder approximation for the bulk sample, the demagnetization factor caused by geometrical shape is about 0.14. For particle samples, the measured demagnetization factor is about 0.27^24^. Accordingly, we evaluated the effect of demagnetization factor on the entropy change *ΔS*, and found the calculated *|ΔS|* value can be enhanced by about ~1.2% and ~2.0% for the bulk and powder samples, respectively, if the corresponding demagnetization field was taking into account. Powder x-ray diffraction (XRD) using Cu Kα radiation was adopted to analyze the structure. We chose typical samples and roughly estimated the grain size from the peak widening of XRD patterns. The obtained grain size is about 80 nm and 10 nm for the particles P4(5~10mm) and P6(1~3mm) of Mn_0.82_Fe_0.18_NiGe, respectively. While for the bulk, the grain size is about 5 μm under SEM.

## Additional Information

**How to cite this article**: Wu, R. *et al*. Critical dependence of magnetostructural coupling and magnetocaloric effect on particle size in Mn-Fe-Ni-Ge compounds. *Sci. Rep.* **6**, 20993; doi: 10.1038/srep20993 (2016).
