# Corrigendum: Critical dependence of magnetostructural coupling and magnetocaloric effect on particle size in Mn-Fe-Ni-Ge compounds

**DOI:** 10.1038/srep25021

**Published:** 2016-04-29

**Authors:** Rongrong Wu, Feiran Shen, Fengxia Hu, Jing Wang, Lifu Bao, Lei Zhang, Yao Liu, Yingying Zhao, Feixiang Liang, Wenliang Zuo, Jirong Sun, Baogen Shen

Scientific Reports 6: Article number: 20993; 10.1038/srep20993 published online: 02172016; updated: 04292016.

In this Article, all instances of the unit “mm” should read “μm”.

